# Mouse Models of Alzheimer’s Disease

**DOI:** 10.3389/fnmol.2022.912995

**Published:** 2022-06-21

**Authors:** Miyabishara Yokoyama, Honoka Kobayashi, Lisa Tatsumi, Taisuke Tomita

**Affiliations:** Laboratory of Neuropathology and Neuroscience, Graduate School of Pharmaceutical Sciences, The University of Tokyo, Tokyo, Japan

**Keywords:** Alzheimer’s disease, β-amyloid, tau, therapeutics, mouse model

## Abstract

Alzheimer’s disease (AD) is a neurodegenerative disorder characterized by memory loss and personality changes, eventually leading to dementia. The pathological hallmarks of AD are senile plaques and neurofibrillary tangles, which comprise abnormally aggregated β-amyloid peptide (Aβ) and hyperphosphorylated tau protein. To develop preventive, diagnostic, and therapeutic strategies for AD, it is essential to establish animal models that recapitulate the pathophysiological process of AD. In this review, we will summarize the advantages and limitations of various mouse models of AD, including transgenic, knock-in, and injection models based on Aβ and tau. We will also discuss other mouse models based on neuroinflammation because recent genetic studies have suggested that microglia are crucial in the pathogenesis of AD. Although each mouse model has its advantages and disadvantages, further research on AD pathobiology will lead to the establishment of more accurate mouse models, and accelerate the development of innovative therapeutics.

## Introduction

**Figure 1:**
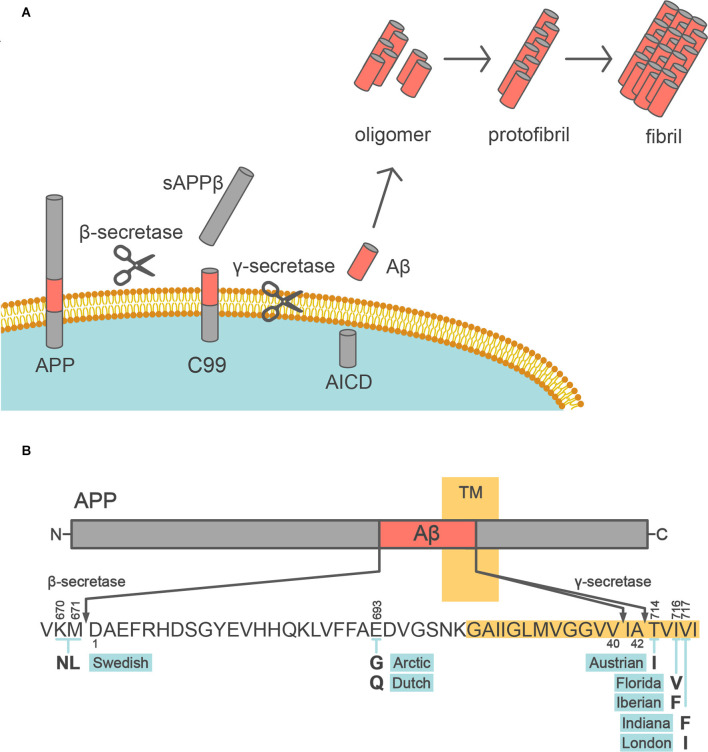
Aβ production from APP. **(A)** Schematic depiction of the Aβ production pathway. APP is sequentially cleaved by β- and γ-secretases to release Aβ into the extracellular space. Aβ monomer is prone to aggregate, forming oligomers, protofibrils, and fibrils. **(B)** Diagram of the APP (above) and sequence of Aβ (below). The transmembrane segment (TM) is highlighted in yellow. The bold letters below the Aβ sequence indicate the missense FAD mutations within the Aβ sequence. The above and below the number of the Aβ sequence follow the numbering of the longest isoform of APP and Aβ amino acid sequence, respectively.

Dementia is defined clinically as a decline in memory and impaired thinking ability, which are two domains of cognition. Alzheimer’s disease (AD) is the most common cause of dementia (Knopman et al., 2021). More than 55 million people have dementia worldwide, and the prevalence of dementia is expected to reach 78 million people by 2030^1^. Most AD patients (>99%) have the sporadic form of AD, whereas very few patients (<1%) have familial AD (FAD), which is inherited in an autosomal dominant manner. AD is characterized neuropathologically by two types of depositions; i.e., β-amyloid plaques and neurofibrillary tangles. β-amyloid plaques are deposited extracellularly, and consist mainly of β-amyloid protein (Aβ). Aβ is produced from its precursor, known as an amyloid precursor protein (APP), *via* sequential cleavage by the β- and γ-secretases (Figure 1A). Neurofibrillary tangles are intraneuronal aggregates comprised of hyperphosphorylated forms of the microtubule-binding protein tau. Longitudinal studies of biomarker changes in AD patients have demonstrated that Aβ deposition occurs first, followed by the accumulation of tau pathology (Bateman et al., 2012; Fagan et al., 2014). However, these pathologies do not act in isolation, and rather form a continuum (Jack et al., 2018). The deposition of Aβ and tau is spatially and temporally associated with the progression of AD.

### Amyloid-β Peptide in the Pathogenesis of Alzheimer’s Disease

Aβ was first isolated from the cerebrovascular amyloid of AD patients in 1984 (Glenner and Wong, 2012) and recognized as the main component of senile plaque. Aβ spontaneously aggregates into β-sheet structures in the form of higher-order oligomers, protofibrils, and fibrils. Numerous studies on human AD biomarkers have demonstrated that the cerebral Aβ deposition begins many years before other AD-associated changes [i.e., increased tau in the cerebrospinal fluid (CSF), decreased cerebral glucose metabolism, brain atrophy, and clinical dementia]. Aβ pathology is considered to occur in stereotypical spatial patterns or topographies throughout the disease course (Thal et al., 2002), whereas recent studies demonstrated that FAD mutation types affect the patterns of Aβ accumulation (Chhatwal et al., 2022).

Aβ is produced by the two-step cleavage of APP by β-secretase and γ-secretase (Kikuchi et al., 2017; Figure 1A). First, APP is processed within the ectodomain by β-site APP-cleaving enzyme 1 (BACE1) as the β-secretase, yielding secreted sAPPβ and the C-terminal fragment C99. Then, C99 is subjected to intramembrane proteolysis by γ-secretase, which releases Aβ. BACE1 is a single-span membrane-anchored aspartic protease, which is mainly expressed in neurons. γ-Secretase is a complex of four protein subunits, namely, presenilin 1 (PSEN1) or PSEN2, nicastrin, presenilin enhancer 2, and anterior pharynx-defective 1 (Takasugi et al., 2003). γ-Secretase generates heterogeneity in the C-terminal length of Aβ, by primarily producing Aβ40 and Aβ42. Aβ40, which is a 40 amino acid peptide, is the most common form of Aβ in humans. Aβ42 differs from Aβ40 only in that it has two extra amino acid residues at the C-terminus (isoleucine-alanine) but is the most toxic and aggregation-prone Aβ species and is the main species deposited in the brains of AD patients (Jarrett et al., 1993; Iwatsubo et al., 1994; Figure 1B). On the other hand, a large variety of N-terminally truncated or elongated Aβ peptides, known as Aβ-related peptides, have been detected in human brains, CSF, and plasma, with the development of mass spectrometry techniques (Wang et al., 1996; Moore et al., 2012; Kaneko et al., 2014). Although the physiological functions of these peptides, including Aβ, remain incompletely understood, the combination of APP669–711 [a.k.a. Aβ (−3)–40, an Aβ-related peptide], Aβ42, and Aβ40 in human plasma can be a surrogate biomarker of cerebral Aβ accumulation (Kaneko et al., 2014; Nakamura et al., 2018).

### Tau in the Neurodegenerative Diseases

**Figure 2:**
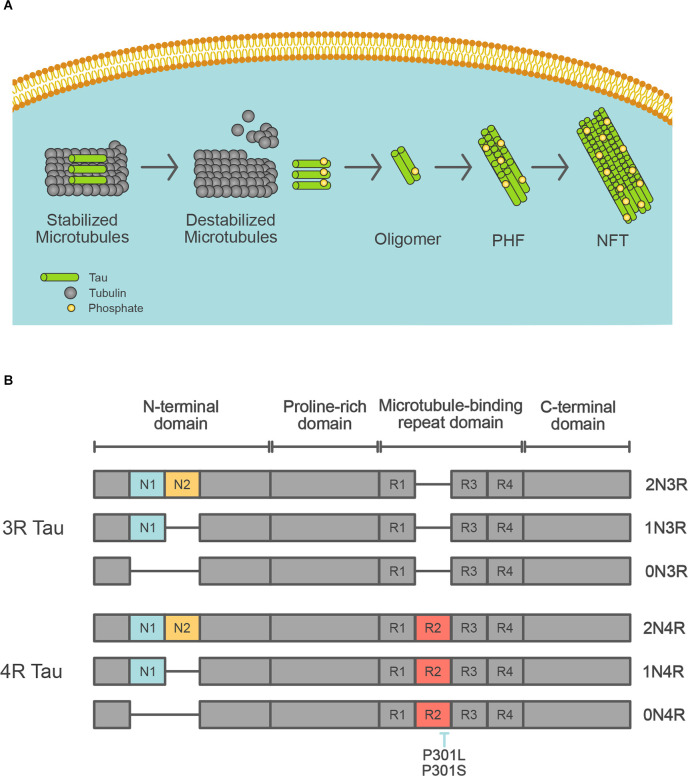
Tau isoforms in the human brain. **(A)** Schematic depiction of the formation of neurofibrillary tangles. Tau protein control stabilization of microtubules by kinases. Phosphorylated tau leads to microtubule disassembly. Irregular hyperphosphorylation of tau proteins results in the generation of insoluble tau oligomers, which then accumulate to form PHF, then NFT. **(B)** Schematic diagram of tau isoforms that are expressed in the adult human brain. *MAPT* gene encodes tau protein. The six tau isoforms are generated by splicing of exons 2, 3, and/or 10 that encode for N1 (blue), N2 (yellow), and R2 (red), respectively. The inclusion of exon 10 results in the generation of mRNA encoding 4R tau. The location of missense *MAPT* mutations utilized in the transgenic mice is shown.

Tau was first described as a major component of neurofibrillary tangles (NFTs) in 1986 (Grundke-Iqbal et al., 1986; Kosik et al., 1986; Nukina and Ihara, 1986). Tau pathology spreads throughout the AD brain in a stereotypical pattern across neural circuits throughout the brain (Braak and Braak, 1991; Jack et al., 2018). The progression of tau pathology is associated with neuronal death (Gómez-Isla et al., 1997). NFTs consist of abundant intracellular paired helical filaments (PHF) whose main constituent is tau (Figure 2A). Aggregated tau is deposited in a variety of neurodegenerative diseases, not only AD, and these diseases are collectively referred to as tauopathy. The tau protein is encoded by the *MAPT* gene on chromosome 17, which contains 16 exons. Based on its function, the tau protein is divided into the following four domains: N-terminal domain, a proline-rich domain, microtubule-binding repeat domain, and C-terminal domain, and is alternatively spliced at the N-terminal domain and microtubule-binding repeat domain to form six isoforms (0N3R, 0N4R, 1N3R, 1N4R, 2N3R, and 2N4R; Figure 2B). Depending on the presence of the repeat domain, tau proteins become either 3R or 4R tau. In the adult human brain, both 3R and 4R tau are expressed at approximately equal levels, but the 3R/4R tau ratio is altered in patients with most of the tauopathies. Tau proteins undergo several different post-translational modifications, including acetylation, glycosylation, nitration, O-GlcNAcylation, phosphorylation, SUMOylation, truncation, and ubiquitination (Wesseling et al., 2020). Tau can be phosphorylated at 85 different residues, and abnormally hyperphosphorylated tau (p-tau) is a key component of neurofibrillary tangles. Some phosphorylation sites of tau (threonine residues at 181, 217, and 231) are specifically phosphorylated in AD patients, and CSF and plasma tau are useful biomarkers of AD pathology (Barthélemy et al., 2020; Janelidze et al., 2020).

### Gene Mutations Linked to Familial Alzheimer’s Disease

As Down syndrome, in which patients demonstrate the same neuropathological changes as AD patients (i.e., senile plaques and neurofibrillary tangles), is caused by a trisomy of chromosome 21, which is where *APP* is located, the association between the *APP* gene and AD has attracted much attention. The first FAD mutation identified as a cause of autosomal-dominant AD was in the *APP* gene, and mutations were subsequently identified in *PSEN1* and *PSEN2* (Goate et al., 1991; Levy-Lahad et al., 1995; Rogaev et al., 1995; Sherrington et al., 1995). In addition to point mutations, an increased copy number of the *APP* gene also causes AD (Rovelet-Lecrux et al., 2006). No FAD mutations have been found in the *MAPT* gene, but mutations in *MAPT* cause familial frontotemporal dementia (FTD) and several other tauopathies (Iqbal et al., 2016; Figure 2B). The most common FTD mutations are P301L and P301S, which lead to tau aggregation. Based on these mutations, mouse models with aggregated tau pathology have been generated. The major FAD mutations are summarized in Tables s 1–3, with a full and more updated version available on the Alzforum website^2^.

**Table 1:** Representative FAD-linked mutations in APP gene used in model mice.

**Name**	**Mutation**	**Effect on Aβ**	**Model mouse**
Swedish	KM670/671NL	Increased total Aβ; unchanged Aβ42/Aβ40 ratio	Tg2576, APP23, J20, TgCRND8, APPswe/PSEN1dE9, 5xFAD, A7, NL-G-F
Arctic	E693G	Arctic Aβ40 forms protofibrils at an increased propensity and faster rate	NL-G-F
Dutch	E693Q	Dutch Aβ increases aggregation rates and fibril formation	-
Austrian	T714I	Increased Aβ42/Aβ40	A7
Florida	I716V	Increased Aβ42(43)/Aβ40	5xFAD
Iberian	I716F	Increased Aβ42/Aβ40 ratio	NL-G-F
Indiana	V717F	Increased Aβ42/Aβ40	PDAPP. J20. TgCRND8
London	V717I	Increased Aβ42/Aβ40	5xFAD

**Table 2:** Representative FAD-linked mutations in PSEN1 used in model mice.

**Mutation**	**Phenotype**	**Mouse model**
M146L	Increased Aβ42/Aβ40 ratio	5xFAD
M146V	Increased Aβ42/Aβ40 ratio	3xTg
L286V	Increased Aβ42/Aβ40 ratio	5xFAD
ΔE9	Increased Aβ42/Aβ40 ratio	APPswe/PSEN1dE9

**Table 3:** Representative APP model mice.

**Model mouse**	**Gene (Mutation)**	**Promoter**	**Aβ pathology**	**Tau pathology**	**Neuronal loss**	**Cognitive impairment**
PDAPP	*APP* (V717F)	PDGF-β promoter	+ (6 mo~)	-	-	+ (3 mo~)
Tg2576	*APP* (KM670/671NL)	hamster prion promoter	+ (11 mo~)	-	-	+ (6 mo~)
APP23	*APP* (KM670/671NL)	Thy1 promoter	+ (6 mo~)	-	+ (12 mo~)	+ (3 mo~)
J20	*APP* (KM670/671NL, V717F)	PDGF-β promoter	+ (8 mo~)	-	+ (3 mo~)	+ (4 mo~)
TgCRND8	*APP* (KM670/671NL, V717F)	hamster prion promoter	+ (3 mo~)	-	+ (6 mo~)	+ (3 mo~)
PS2APP	*APP* (KM670/671NL) *PSEN2* (N141I)	APP: Thy1.2 promoter *PSEN2*: mouse prion promoter	+ (9 mo~)	-	unknown	+ (8 mo~)
APPswe/PSEN1dE9 (APP/PS1)	*APP* (KM670/671NL) *PSEN1* (delta9)	mouse prion promoter	+ (6 mo~)	-	+ (8 mo~)	+ (12 mo~)
Tg-ArcSwe	*APP* (KM670/671NL, E693G)	Thy1 promoter	+ (5 mo~)	-	-	+ (4 mo~)
5xFAD	*APP* (KM670/671NL, I716V, V717I) *PSEN1* (M146L, L286V)	Thy1 promoter	+ (2 mo~)	-	+ (6 mo~)	+ (4 mo~)
A7	*APP* (KM670/671NL, T714I)	Thy1.2 promoter	+ (9 mo~)	unknown	unknown	unknown
NL-G-F	humanized Aβ sequence (KM670/671NL, I716F, E693G)	Endogenous promoter (knock-in model)	+ (2 mo~)	-	-	+ (6 mo~)

FAD mutations in *APP* are named according to the region of origin of the affected family, and mainly occur at its two cleavage sites, leading to Aβ overproduction or increased aggregation (Figure 1B). The Swedish mutation (K670N/M671L) is at the β-secretase cleavage site, and results in increased β-secretase mediated cleavage leading to enhanced production of both Aβ40 and Aβ42. The Indiana (V717F), London (V717I), and other mutations at the γ-cleavage sites promote the production of Aβ42, which is more toxic than Aβ40. *APP* mutations within Aβ, such as the Arctic (E693G) and Dutch (E693Q) mutations, increase Aβ aggregation and promote the formation of stable oligomers and protofibrils. Moreover, almost all *PSEN1* and *PSEN2* mutations in FAD cause an increase in the Aβ42/Aβ40 ratio (Borchelt et al., 1996; Duff et al., 1996; Citron et al., 1997; Tomita et al., 1997; Table 2). Finally, a protective variant of APP (A673T) against AD and age-associated cognitive decline was identified (Jonsson et al., 2012). Of note, this variant reduced β-secretase cleavage and total Aβ production, supporting the notion that brain Aβ deposition is a central mechanism in the pathogenesis of AD (Hardy and Selkoe, 2002; Selkoe and Hardy, 2016; Karran and De Strooper, 2022).

## Aβ Deposited Mouse Models Based on APP Mutations

Wild-type mice do not develop senile plaques or neurofibrillary tangles even in old age, and hence cannot be used as model animals for AD. Based on the genetic findings that almost all FAD-linked mutations are associated with alterations of Aβ production/aggregation, advances in genetic engineering technology have enabled the development of model mice using the *APP* and *PSEN1* genes (Tables s 1; 2). In the previous few decades, several model mice with cerebral Aβ deposition have been developed by the overexpression or knock-in of the human *APP* gene carrying FAD-linked mutations. In particular, some model mice have been widely utilized in many studies to clarify the pathological process of AD, and to test the therapeutic effects of potential interventions ( Table 3).

### PDAPP Mice

PDAPP was the first AD mouse model that was found to have Aβ deposition and expresses the human *APP* gene with the Indiana mutation (V717F) driven by the platelet-derived growth factor (PDGF)-β promoter (Games et al., 1995). PDAPP mice begin to develop human Aβ deposits in the cerebral cortex at approximately 6–9 months of age, together with increased gliosis in an age-dependent manner. Synaptic and dendritic densities are reduced in the molecular layer of the hippocampal dentate gyrus by 8 months of age. By 3 months of age, PDAPP mice show memory deficits, which also progress in an age-dependent manner.

### Tg2576 Mice

Tg2576 is one of the most widely used AD mouse models, which overexpresses the human *APP* gene with the Swedish mutation (KM670/671NL) under the hamster prion promoter (Hsiao et al., 1996). Tg2576 mice show amyloid plaque formation from 11 to 13 months of age, and microglial activation in or around plaques in all the regions of the neocortex and hippocampus from 10 to 16 months of age. Although no neuronal loss has been detected, some memory functions such as spatial alternation and spatial reference learning are found to be impaired by 10 months of age (Frautschy et al., 1998).

### APP23 Mice

APP23 mice express the human *APP* gene with the Swedish mutation (KM670/671NL) under the human Thy1 promoter (Sturchler-Pierrat et al., 1997). APP23 mice develop Aβ deposition in the brain starting from 6 months of age and these deposits increase with age in size and number, leading to the occupation of a substantial area in the neocortex and hippocampus by 24 months of age. A massive glial response can be detected in brain areas with Aβ plaques. Neuronal loss adjacent to Aβ deposition is apparent in 12-month-old mice. Cognitive decline in this model starts from 3 months of age, preceding amyloid deposition (Van Dam et al., 2003).

### J20 Mice

J20 mice overexpress the human *APP* gene with two FAD-linked mutations, the Swedish mutation (KM670/671NL) and the Indiana mutation (V717F), under the PDGF-β promoter (Mucke et al., 2000). J20 mice develop diffuse amyloid deposition at 5–7 months of age and high hippocampal plaque loads at 8–10 months of age. An increased number of activated astrocytes and microglia are observed in the hippocampus at 6–9 months of age (Wright et al., 2013). Preceding amyloid deposition, neuronal loss and some cognitive deficits appear in this model.

### TgCRND8 Mice

TgCRND8 mice overexpress the human *APP* gene harboring two FAD-linked mutations, the Swedish mutation (KM670/671NL) and the Indiana mutation (V717F), under the hamster prion promoter (Chishti et al., 2001). Deposition of Aβ in this mouse model starts from 3 months of age and becomes more extensive with age. Activated microglia appear accompanied by amyloid plaque accumulation at 3 months of age, shortly followed by a robust astrocytic response (Dudal et al., 2004). The acquisition and learning reversal of spatial information are impaired by 3 months of age.

### PS2APP Mice

PS2APP mice were created by crossing two single transgenic models, APPswe mice and PS2 (N141I) mice (Richards et al., 2003). This model overexpresses the human *APP* gene carrying the Swedish mutation (KM670/671NL) and the human *PSEN2* gene with N141I mutation under Thy1.2 promoter and mouse prion promoter, respectively. Amyloid plaques begin to appear in the subiculum and frontolateral cortices at 9 months of age, spreading to not only most of the neocortex, hippocampal formation, and amygdala but also to thalamic and pontine nuclei by 13–17 months. Gliosis is observed around Aβ plaques at 9 months. Cognitive functions are impaired in 8-month-old mice.

### APPswe/PSEN1dE9 (APP/PS1) Mice

APPswe/PSEN1dE9 mice were developed by coinjecting two vectors driven by the mouse prion promoter. One vector encodes the *APP* gene harboring the Swedish (KM670/671NL) mutation and the other encodes the FAD-linked *PSEN1* gene without exon 9 (dE9; Jankowsky et al., 2004). APPswe/PSEN1dE9 mice show Aβ deposition from 6 months of age and it becomes abundant in the hippocampus and cortex by 9 months. These deposits are surrounded by reactive astrocytes (Kamphuis et al., 2012). Performance in the Morris water maze is impaired at 12 months of age (Lalonde et al., 2005).

### Tg-ArcSwe Mice

Tg-ArcSwe mice overexpress the human APP gene harboring two FAD-linked mutations, the Arctic mutation (E693G) and the Swedish mutation (KM670/671NL), driven by Thy1 promoter (Lord et al., 2006). Extracellular amyloid deposition is observed at 5–6 months of age and becomes quite frequent in the cerebral cortex, subiculum, and hippocampus at 9 months. These amyloid plaques are surrounded by marked astrogliosis. Spatial learning performance is impaired in 4-month-old mice (Lord et al., 2009). Neuronal loss was not detected in this mouse model.

### 5xFAD Mice

This mouse model was generated by coexpressing the following five FAD mutations in APP/PS1 double-transgenic mice: the Swedish (KM670/671NL), Florida (I716V), and London (V717I) mutations in the *APP* gene, and the M146L (A > C) and L286V mutations in the *PSEN1* gene, causing the accelerated formation of Aβ plaques and very high cerebral Aβ42 levels (Oakley et al., 2006). Aβ plaques in the brain appear at 2 months of age and increase in an age-dependent manner, becoming saturated by 6–9 months of age, together with robust gliosis. Regarding cognitive impairment, spatial working memory in the Y-maze, associative memory in the trace fear conditioning test, and spatial memory in the Morris water maze are all impaired by 6 months of age.

### A7 Mice

A7 mice overexpress APP harboring the Swedish (KM670/671NL) and Austrian (T714I) mutations under the control of the Thy1.2 promoter (Yamada et al., 2009). A7 mice demonstrate progressive amyloid deposition in the cerebral cortex from 9 to 12 months. Other pathologies than Aβ deposition have not been reported so far.

### App^NL-G-F^ Knock-In Mice

*App^NL-G-F^* knock-in mice were developed to increase the amount of Aβ42 without overexpressing the human *APP* gene. The Aβ sequence was humanized, and the Swedish (KM670/671NL), Arctic (E693G), and Iberian (I716F) mutations were introduced into the *App* mouse gene. Cortical Aβ deposition begins by 2 months, and is almost saturated together with robust gliosis by 7 months (Saito et al., 2014). Performance on the spatial alternation task, learning ability, and spatial memory retention is impaired by 6 months of age. *App^NL-G-F^* knock-in mice does not show any neuronal loss.

## Tauopathy Mouse Models Based on Mapt Mutations Or Seed Injection

Although familial mutations in the *MAPT* gene have not been identified in FAD patients, the identification of familial FTD-linked mutations has facilitated the development of tauopathy mouse models (Table 4). There are broadly two types of tauopathy model mice, i.e., genetically modified models, and tau seed-injection models. These models are diverse regarding the genetic engineering method (transgenic or knock-in), expressed isoforms of tau (0N4R, 1N4R, etc.), tau expression level, and brain regions in which the introduced tau is highly expressed (Table 5).

**Table 4:** Representative missense mutations linked to tauopathies used in model mice.

**Mutation**	**Phenotype**	**Mouse model**
P301L	Accelerated formation of aggregated tau	JNPL3, rTg4510, 3xTg
P301S	Accelerated formation of aggregated tau	PS19

**Table 5:** Representative tau model mice.

**Model mouse**	**Gene (Mutation)**	**Promoter**	**Aβ pathology**	**Tau pathology**	**Neuronal loss**	**Cognitive impairment**
JNPL3	*MAPT* 0N4R (P301L)	mouse prion promoter	-	+(5 mo~)	+(5 mo~)	unknown
PS19	*MAPT* 1N4R (P301S)	mouse prion promoter	-	+(6 mo~)	+(9 mo~)	+(6 mo~)
rTg4510	*MAPT* 0N4R (P301L)	CaMKII promoter	-	+(4 mo~)	+(6 mo~)	+(6 mo~)
3xTg	*APP* (KM670/671NL) *MAPT* 0N4R (P301L) *Psen1* (M146V knock-in)	Thy1.2 promoter	+(6 mo~)	+(12 mo~)	unknown	+(4 mo~)
MAPT knock-in	*MAPT* gene	Endogenous promoter (knock-in model)	-	-	-	-
NL-G-F/MAPT double knock-in	humanized Aβ sequence (KM670/671NL, I716F, E693G) *MAPT* gene	Endogenous promoter (knock-in model)	+	-	-	unknown

### JNPL3 Mice

JNPL3 mice were the first mouse model established to carry a *MAPT* mutation (Lewis et al., 2000). They express human 0N4R tau with the P301L mutation, driven by the mouse prion protein promoter. This model is particularly characterized by strong motor and behavioral deficits, which may be caused by neuropathological changes in the spinal cord. At 4.5 months, they show tangle pathology in the diencephalon, brainstem, cerebellar nuclei, and spinal cord, as well as motor impairment. Neuronal loss occurs from 10 months of age, particularly in the spinal cord. Astrogliosis in the brainstem, diencephalon, and basal telencephalon is seen from 10 months of age.

### PS19 Mice

PS19 mice express human 1N4R tau with the P301S mutation, driven by the mouse prion promoter (Yoshiyama et al., 2007). PS19 mice are characterized by early synapse loss and microglial activation before neurofibrillary tangle pathology and neuronal loss. PS19 mice develop microgliosis as early as 3 months of age. Neurofibrillary tangle-like inclusions are seen from 6 months, and are widespread throughout the neocortex, amygdala, hippocampus, brain stem, and spinal cord. Neuronal loss and brain atrophy are seen by 8 months, principally in the hippocampus and also spreading to the neocortex and entorhinal cortex. Astrogliosis is robustly increased at 6-months old compared with at 3 months old. Motor deficits, such as clasping and limb retraction when lifted by the tail are seen, followed by limb weakness. These deficits progress to paralysis at 7–10 months, and about 80% of PS19 mice die by 12 months of age (Yoshiyama et al., 2007).

### rTg4510 Mice

rTg4510 mice express human 0N4R tau with the P301L mutation, and are unique in that transgene expression can be regulated (Santacruz et al., 2005). These mice carry the mutant *MAPT* gene downstream of a tetracycline operon-responsive element, and a tetracycline-controlled transactivator is expressed under the control of the calcium/calmodulin-dependent protein kinase II alpha (CaMKIIα) promoter. The transgene expression is inactivated by the administration of the tetracycline analog doxycycline. The expression level of this mutant human tau is 13-fold higher than that of the endogenous mouse protein. However, the transgenes disrupt six endogenous mouse genes, including Fibroblast Growth Factor 14 which might affect the neuropathological and neurodegenerative phenotypes observed in rTg4510 mice (Gamache et al., 2019). Argyrophilic tangle-like inclusions are observed in the cortex and hippocampus by 5.5 months (Santacruz et al., 2005). Neuronal loss starts from 5.5 months in CA1 hippocampal neurons, and gross atrophy of the forebrain was seen by 10 months of age. Retention of spatial memory is impaired from 2.5 to 4 months of age. Notably, neuronal death ceases and the decline in cognitive function is arrested or even reversed following transgene suppression with dox (Santacruz et al., 2005).

### 3xTg Mice

These mice were established by the coinjection of two AD-associated human genes carrying FAD-linked mutations [i.e., *APP* with the Swedish mutation (KM670/671NL), and *MAPT* with the P301L mutation] into the embryo of homozygous *Psen1* M146V knock-in mice (Oddo et al., 2003). The expression of both transgenes is regulated by the mouse Thy1.2 promoter. These mice develop progressive neuropathology, including intracellular and extracellular Aβ deposits and conformationally altered phosphorylated tau aggregates.

### Human MAPT Knock-In Mice

These mice carry the humanized WT *MAPT* gene, instead of the endogenous murine *MAPT* gene (Saito et al., 2019). They express all six isoforms of human tau, with the level of 4R mRNA being approximately 70% of that of 3R mRNA. *App^NL-G-F^/MAPT* double knock-in mice lack neurofibrillary tangles and do not show neurodegeneration, although they develop amyloid plaques and Aβ-associated pathologies. Notably, tau humanization accelerates the cell-to-cell propagation of AD patient-brain derived tau both in the absence and presence of Aβ deposition.

### Seed Injection Models

The first seed injection model was established in 2009 (Clavaguera et al., 2009), and transmission and diffusion of the tau were demonstrated. Seed injection models are created by injecting brain lysates from the brains of AD patients or model mice, or recombinant tau into mouse brains. In these models, the spreading of pathological tau occurs *via* synaptic connections rather than between proximal cells (Ahmed et al., 2014). Seed injection models identified several important elements that affect tau pathology, including age, Aβ, oligomerization, or splicing isoform of tau (Jackson et al., 2016; He et al., 2018; Hosokawa et al., 2021; Nies et al., 2021).

## Mouse Models Modified TREM2 Signaling

Triggering receptor expressed on myeloid cells 2 (TREM2), which is expressed in myeloid cells including microglia in the brain, is an AD risk gene and the R47H variant shows a robust odds ratio (Guerreiro et al., 2013; Jonsson et al., 2013) and is implicated in the development of late-onset AD (Ulland and Colonna, 2018). Microglia have been shown to respond to Aβ accumulation and neurodegenerative lesions, progressively acquiring a unique transcriptional and functional signature (Keren-Shaul et al., 2017; Krasemann et al., 2017). These microglia attenuate the progression of neurodegeneration in some mouse models, but inappropriate microglial activation accelerates neurodegenerative disease in other models. The loss of TREM2 in APP model mice was found to facilitate the accumulation and spread of tau (Yuan et al., 2016; Leyns et al., 2019), suggesting the important role of TREM2 in the pathological connection between Aβ and tau. Note that marked species-specific differences in TREM2-mediated signaling have been identified, and hence further investigation of the TREM2 pathway in various species is required in the future (Zhou et al., 2020).

## Preclinical Analyses of Potential AD Therapies Using Model Mice

The safety and efficacy of any drug must be confirmed using animal models in a preclinical study before it proceeds to clinical studies. As described above, there are various types of AD mouse models, and the model that is used for a preclinical study has a substantial effect on the outcomes.

### Secretase Inhibitors and Modulators

The development of secretase inhibitors has been attempted, aiming to treat AD by reducing Aβ production. In the development of semagacestat, the first γ-secretase inhibitor tested in a phase III clinical trial, pharmacodynamics studies were performed using PDAPP mice (Panza et al., 2010). However, semagacestat caused irreversible symptoms, such as the worsening of cognitive function, skin cancer, and infections in PDAPP mice, leading to the suspension of the phase III trial and the further development of this potential drug (Doody et al., 2013). Furthermore, Tg2576 mice were utilized in the development as well as comparison of other γ-secretase inhibitors, such as avagacestat (Mitani et al., 2012). However, phase II trials of avagacestat were discontinued owing to gastrointestinal and dermatological adverse events that occurred in these mice (Coric et al., 2015).

BACE1, which is responsible for the N-terminal processing of Aβ, has been investigated as a therapeutic target molecule. Various BACE1 inhibitors, such as verubecestat, umibecestat, and atabecestat have been developed, and their efficacy and pharmacodynamics have been investigated in animal models (Kennedy et al., 2016; Neumann et al., 2018; Koriyama et al., 2021). However, none of them have shown therapeutic effects in clinical trials (Egan et al., 2018, 2019; Novak et al., 2020). Although secretase inhibitors may have therapeutic potential as a maintenance therapy after plaque removal or as a preventive drug (McDade et al., 2021), to date, none of the BACE1 inhibitors have passed clinical trials and become available as therapeutic agents.

### Immunotherapies Targeting Amyloid-β Peptide

The use of anti-Aβ antibodies to prevent the formation of Aβ plaques has been investigated since the 1990s. In 1996, it was shown that two monoclonal antibodies, AMY-33 and 6F/3D, inhibit Aβ aggregation *in vitro*, indicating the possibility that monoclonal antibodies that prevent Aβ aggregation may exist *in vivo* (Solomon et al., 1996). Since then, Schenk et al. showed that immunization of PDAPP mice with human Aβ42 prevented the formation of Aβ plaques, neuritic dystrophy, and astrogliosis, and markedly reduced the extent and progression of AD pathology (Schenk et al., 1999). In addition, peripherally administered antibodies against Aβ were found to enter the central nervous system and significantly trigger the clearance of Aβ accumulation in PDAPP mice (Bard et al., 2000). Moreover, it was reported that immunization with Aβ reduced amyloid plaque formation and also prevented cognitive deficits in Aβ deposited mouse models (Janus et al., 2000; Morgan et al., 2000). These preclinical results prompted researchers to consider anti-Aβ immunotherapy as a potential treatment of AD. Pre-aggregated synthetic Aβ42 (AN-1792) combined with the immunogenic adjuvant QS-21, the first active immunotherapy strategy for AD, has entered clinical trials. However, 6% of the patients treated with AN-1792 developed symptoms of meningoencephalitis in the phase IIa trial initiated in 2001, and the trial was discontinued in 2003 (Gilman et al., 2005). Nicoll et al. reported that the vaccinated patients showed very few Aβ plaques in the brain parenchyma, and small Aβ aggregates were found associated with microglia (Nicoll et al., 2003).

The activation of passive immunity by the administration of anti-Aβ monoclonal antibodies has also been investigated (Wilcock et al., 2004). Passive immunotherapy has several advantages, such as the ability to control the number of antibodies and to prevent the occurrence of unexpected effects caused by high antibody specificity. At present, lecanemab, gantenerumab, donanemab, and aducanumab have reached the final stage of development. All of these potential drugs have undergone preclinical studies using mouse models. Lecanemab is an anti-protofibril antibody, and is a humanized version of the mouse antibody mAb158. The administration of mAb158 to aged Tg-ArcSwe mice reduced Aβ protofibrils without changing insoluble Aβ levels (Tucker et al., 2015). Gantenerumab recognizes the N-terminal and central regions of Aβ. Gantenerumab bound to cerebral Aβ and enhanced the removal of small Aβ plaques by recruiting microglia, and prevented new plaque formation in PS2APP mice (Bohrmann et al., 2012). Donanemab is an antibody against Aβp3-x, which is a pyroglutamate form of Aβ, and aggregates early in the deposition cascade (Saido et al., 1995). Donanemab is derived from the murine antibody mE8-IgG2, and was shown to bind specifically to Aβ plaques and to remove existing plaques in aged PDAPP mice (DeMattos et al., 2012). Aducanumab is a human monoclonal antibody that binds aggregated forms of Aβ, but not Aβ monomers. Aducanumab bound the cerebral Aβ deposits and reduced Aβ burden in a dose-dependent manner when administered to aged Tg2576 and APP23 mice (Sevigny et al., 2016; Uhlmann et al., 2020). These findings suggest that Aβ deposited mouse models based on APP mutations are useful for the development of anti-Aβ deposition therapies, although the effects of these therapies on human cognitive function remain controversial.

### Immunotherapies Targeting Tau

Antibodies bind extracellular molecules and do not penetrate the cell membrane. Thus, tau had not initially been considered as a target molecule for immunotherapy. However, after the identification of extracellular tau (eTau), which is involved in the propagation of tau pathology in model mice (Yamada, 2017), researchers have considered immunotherapy against tau as a potential treatment for AD. As in Aβ, many tau-targeting drugs aim for the clearance of tau. Anti-tau antibodies can be classified by their epitopes; i.e., antibodies against pan-tau, p-tau, and tau with a pathological conformation (Congdon and Sigurdsson, 2018). Semorinemab was the first anti-tau antibody to be developed, which binds to the N-terminus of all six isoforms of human tau, and both monomeric and oligomeric eTau. Treatment for 13 weeks with semorinemab dose-dependently reduced brain pathology in tau P301L transgenic mice (expressing human tau P301L under the Thy1 promoter; Ayalon et al., 2021). Gosuranemab also targets the N-terminus of eTau. Gosuranemab neutralized the toxicity of eTau in rTg4510 mice (Sopko et al., 2020). However, these antibodies did not improve outcomes in clinical trials, probably owing to the inability of tau N-terminus-targeting antibodies to inhibit the spread of tau in humans. The JNJ-63733657 antibody targets the microtubule-binding region of tau, and has a high affinity for p-tau. This antibody is thought to target aggregated tau more potently than antibodies targeting the N-terminus. Zagotenemab is a humanized anti-tau antibody derived from the MCI-1 antibody that specifically targets soluble tau aggregates. The peripheral administration of MCI-1 substantially reduced tau pathology in the JNPL3 mouse model (Chai et al., 2011). However, the development of zagotenemab has been halted because of its lack of efficacy in humans. The lack of success of anti-tau antibodies in clinical trials may be owing to differences in the mechanisms of tau pathology propagation in model mice and humans, or because the approach of targeting eTau may not be effective against the tauopathies.

### Antisense Oligonucleotides

In recent years, with the development of modified nucleic acids and drug delivery technologies, antisense nucleotides (ASOs) have been used as drugs for the treatment of neurodegenerative diseases. BIIB080 was the first ASO targeting tau expression to enter clinical trials. This ASO inhibits the translation of tau mRNA into protein, and treatment of PS19 model mice with BIIB080 led to the prevention of tau pathology, accompanied by the prevention of hippocampal volume loss, neuronal death, and behavioral deficits (DeVos et al., 2017).

### Inhibitors of Post-translational Modifications of Tau

Post-translational modifications of tau, including hyperphosphorylation and acetylation, interfere with the binding of tauto microtubules, and promote tau misfolding. Thus, targeting any of these modifications, alone or in combination, has the potential to prevent tau aggregation and restore its normal function. Memantine, which is a phosphatase modifier that enhances PP2A (Protein phosphatase 2) activity, improves cognition, and reduces tau pathology in model mice (Chohan et al., 2006). Kinase inhibitors, such as CDK5 inhibitors, attenuated pathology, and neurodegeneration in tauopathy mice (Malhotra et al., 2021). An acetyltransferase inhibitor was shown to rescue tau-induced memory deficits and to prevent hippocampal atrophy in PS19 mice (Min et al., 2015). However, to date, no therapeutic reagent against the post-translational modifications of tau has shown beneficial effects in clinical trials.

### Photo-Oxygenation System as a Potential Treatment for AD

The challenge of immunotherapy using anti-Aβ antibodies is that they do not easily permeate the blood-brain barrier. To overcome this problem, the development of low-molecular-weight therapeutic drugs has been promoted. Photocatalysts activated by light irradiation only when they bind β-sheet structures of amyloid have been developed (Taniguchi et al., 2016; Ni et al., 2018; Suzuki et al., 2019). This photo-oxygenation system successfully inhibited the aggregation of recombinant Aβ and tau *in vitro*. In addition, photo-oxygenation treatment of *App^NL-G-F^* mice decreased cerebral Aβ levels, and facilitated microglial clearance *in vivo* (Nagashima et al., 2021; Ozawa et al., 2021). It is expected that this new, and innovative amyloid-targeting system will greatly influence the establishment of therapeutic interventions for neurodegenerative diseases in the future (Ikeda et al., 2021).

### Identification of Biomarkers of AD

The Aβ-targeting therapies described above are most effective when they are initiated early in the course of AD because cerebral Aβ deposition is the earliest pathology of AD. Thus, the development of biomarkers that can detect an increase in brain Aβ accumulation before the onset of dementia is needed. Common AD biomarkers include Aβ and tau levels in the CSF (Arai et al., 1995; Motter et al., 1995; Kanai et al., 1998). However, the time lag between CSF collection and autopsy in patients has been a big challenge in understanding the association between the progression of brain pathology and biomarker alterations. Thus, model mice have been utilized to analyze the association between biomarkers and brain pathology. A correlation between the decrease in CSF Aβ level and the increase in brain Aβ level has been observed in various mouse models (Kawarabayashi et al., 2001; Maia et al., 2013). In addition, CSF tau levels were increased in a mouse model with Aβ deposition in the brain (Maia et al., 2013). Recently, the novel plasma biomarker APP669–711/Aβ42, which detects the presence of Aβ accumulation in the brain with high sensitivity and specificity was established (Nakamura et al., 2018). Changes in this plasma biomarker have been reproduced in APP/PS1 model (Matsuzaki, Yokoyama, and Tomita, unpublished observations). The analysis of biomarkers in AD mouse models provides a valuable opportunity to clarify the biological mechanism of the biomarkers identified in AD patients, and supports the validity of further biomarker studies *in vivo*.

## Concluding Remarks and Future Prospectives

Elucidation of the molecular pathogenesis of AD based on genetic and biochemical analyses has established Aβ and tau as the two main pathogenic factors. In this regard, the use of genetically engineered mice, as well as mice injected with patient brain-derived taufibrils, has made substantial contributions. These mice have also played important roles in the development of therapeutic strategies and diagnostic biomarkers. While an AD mouse model in which the deposition of both Aβ and tau occurs in a similar manner to that observed in sporadic AD patients has not yet been established, cats, dogs, wolverine, and nonhuman primates spontaneously develop age-dependent AD-like brain pathologies (Nakamura et al., 1995; Roertgen et al., 1996; Vite and Head, 2014). The use of nonhuman primates is especially essential to assess the efficacy and safety of therapeutics for AD (Gandy et al., 2004; Lemere et al., 2004; Britschgi et al., 2009), but it has ethical issues due to their high resemblance. To secure and maintain facilities for breeding and housing is also costly difficult, but conversely, mouse models do not have these disadvantages. We should maximize the advantages of each animal model by clarifying the mechanism in detail with mouse models where genetic intervention is easier and validating them with nonhuman primates, and should minimize animal suffering or discomfort by applying 3Rs principle (Replacement, Reduction, and Refinement). Moreover, further understanding of the pathological process of Aβ and tau deposition, as well as its reproduction in the animals will assist in the development of more accurate disease models, which will narrow the gap between preclinical studies and clinical trials.

## Author Contributions

MY, HK, and LT wrote the draft and revised it. TT was involved in discussing, drafting, and editing the manuscript. All authors contributed to the article and approved the submitted version.

## Conflict of Interest

The authors declare that the research was conducted in the absence of any commercial or financial relationships that could be construed as a potential conflict of interest.

## Publisher’s Note

All claims expressed in this article are solely those of the authors and do not necessarily represent those of their affiliated organizations, or those of the publisher, the editors and the reviewers. Any product that may be evaluated in this article, or claim that may be made by its manufacturer, is not guaranteed or endorsed by the publisher.

## Footnotes

^1^ https://www.alzint.org/resource/world-alzheimer-report-2021/

^2^ https://www.alzforum.org/mutations
